# Occupational hazards and risks among the women in fisher communities in Cox’s Bazar and Chattogram, Bangladesh

**DOI:** 10.1371/journal.pone.0297400

**Published:** 2024-07-19

**Authors:** Charls Erik Halder, Partha Pratim Das, S. M. Tareq Rahman, Liton Chandra Bhoumick, Hamim Tassdik, Md. Abeed Hasan, Sourav Nath Mithun

**Affiliations:** 1 Public Health and Research Division, Bright Bangladesh Forum, Chattogram, Bangladesh; 2 Department of Public Health, State University of Bangladesh, Dhaka, Bangladesh; 3 Department of Public Health, North South University, Dhaka, Bangladesh; 4 UFR Sciences du Vivant, Universite Paris Cite, Paris, France; Bangladesh University of Health Sciences, BANGLADESH

## Abstract

**Background:**

Women in the fisher communities in coastal regions of Bangladesh are engaged in a wide range of fishery activities. However, there is limited evidence available on the occupational hazards and risks experienced by them.

**Method:**

The study was conducted among fishing colonies in Cox’s Bazar and Chattogram districts in Bangladesh. This was a cross-sectional study blending qualitative and quantitative approaches. The qualitative component comprised five focus group discussions to understand the occupational context, hazards, and risks faced by the fisherwomen, informing the questionnaire design for the subsequent survey. The quantitative survey involved a sample of 207 women from fisher communities, gathering socio-demographic information, occupational hazards, risks, and health and safety practices.

**Findings:**

The study found a high occurrence of occupational hazards, health risks and limited availability of preventive measures among the women in fisher communities. Occupational hazards include physical safety hazards such as slippery surfaces and fish cutting instruments, and physical hazards like prolonged sun exposure and noise. Chemical hazards like pesticides and saltwater, ergonomic hazards such as prolonged uncomfortable posture and heavy lifting, and biological hazards including inadequate sanitation facilities were prevalent. The study also identified the potential occupational risks, and the outcomes resulting from the hazards, including injuries (87.44%), musculoskeletal conditions (69.08%), skin diseases/conditions (56.52%), eye complaints (33.82%), severe respiratory distress (24.15%) and high incidence of self-reported communicable diseases. Most women (78.26%) did not use personal protective equipment, and the majority (93.72%) lacked a workplace first aid kit.

**Conclusion:**

The study revealed a high prevalence of occupational hazards and health risks, including injuries and diseases, among Bangladeshi women in fisher communities with insufficient safety measures. Collaboration among government, NGOs, development partners, fisheries stakeholders, and the community is imperative for targeted training, innovative procedures, ergonomic solutions, provision of protective equipment, and advocacy to enhance the well-being of these women.

## Background

Globally, the fisheries and aquaculture sector produce more than two hundred million tonnes of fish, aquatic animals and algae critically contributing to global food security and nutrition [1]. This industry employs over 58.5 million people worldwide, with women making up 21% of those workers [2]. In Bangladesh, around 17 million people depend on fishing, fish farming, fish handling, and fish processing for their sustenance [3]. Marine fishery is a vital component in Bangladesh’s fishing industry because of the country’s extensive coastline and marine zone.

Around 30% of the women in rural and coastal areas of Bangladesh are engaged in the fisheries sector [4]. Women in fisher communities in Bangladesh have a twofold dilemma since patriarchal societal norms limit their activities to the home, but rising poverty and landlessness also force them to look for wage work. Thereby, they are to play both roles, fishing activities as well as traditional household chores. An earlier baseline study in Cox’s Bazar and Chattogram revealed that women in the fisher communities were engaged in a wide range of fisheries activities including fish sorting, grading, cutting, dry fish processing, dry fish paste (*Nappi*) processing, daily labouring in fish farms, transporting, selling fish or fish products, net making, ice processing, fish basket making, and collecting minnow and seashells from the sea shores [5]. Still, traditionally, fishing and related activities in Bangladesh are considered to be the occupation of men in Bangladesh, particularly in coastal regions, and women’s contributions to this sector are grossly neglected.

Globally, people engaged in the fishery sector encounter numerous occupational hazards, including the risk of the boat capsizing, slippery surfaces, unsafe handling of heavyweights, fish bites and stings, snake and insect bites and exposure to physical hazards, like sun, salt water and noise, which may result in various illness and deteriorated health conditions, including injuries, fatalities, skin, respiratory and auditory diseases [6–8], According to the World Health Organisation (WHO) and the International Labour Organisation (ILO), globally, almost 2 million individuals died as a result of occupational hazards in 2016 [9].

The Committee on Fisheries (COFI) declaration for sustainable fisheries and aquaculture emphasized enhancing women’s full access to and equal opportunities in the fisheries sector [10]. To achieve this, occupational safety and health of the women engaged in the fisheries should be a key consideration. There are several studies available both at the country and global level on the occupational hazards encountered by men in the fisheries sector. However, only a few studies [11–13] are available that identified occupational hazards and risk factors of women engaged in fishery activities. In Bangladesh, there is no study available on the occupational hazards of women working in fisheries, except, a qualitative study [14] found that women engaged in fishing with their children possess the risk of drowning since the traditional clothes are not flexible enough for free-movement in the water.

The fishery is one of the oldest and most prevalent occupations in Cox’s Bazar and Chattogram coastal communities [15, 16]. Fishery encompasses a wide range of occupational activities, including production, harvesting, processing, handling, storage and transportation. Traditionally, men and women have different roles, while men are mostly engaged in harvesting, women are mostly engaged in fish processing [5]. Therefore, women may experience distinctive hazards and risks in their occupation, which is vital to reveal to ensure a safe work environment for them. Moreover, it is also essential to understand the measures in place to prevent occupational hazards and risks, such as personal safety training and personal protection equipment, for proper planning of preventive strategies. A few studies in similar settings in developing countries, like India and Kenya, found that women in fisher communities face various occupational hazards such as exposure to smoke, sharp knives, sun rays, heavy loads, cold conditions, spine injuries, and slippery floors, as well as heightened risks of cuts, hand blanching, sunburn, falls, musculoskeletal injuries, pricks from spines, eye and nose irritability [11–13]. However, there is no study available in Bangladesh that explicitly described the occupational hazards and risks encountered by the women in the fisher communities, and the health and safety measures that are in place to mitigate occupational hazards and risks. This study was conducted to investigate the occupational hazards, risks and safety measures among women in fisher communities in Chattogram and Cox’s Bazar districts in Bangladesh. Below were the specific objectives of the study:

1. Identify the socio-demographic profile of women engaged in fishery activities, including age, education, ethnicity and family type.
2. Explore the various occupational hazards faced by women in fisher communities, including physical safety, physical, chemical, ergonomic, and biological hazards.
3. Assess the occupational risks resulting from these hazards, including the incidence of injuries, and related illnesses and health conditions.
4. Explore the status of existing health and safety measures in place to prevent occupational health hazards among women in fisher communities, especially, personal protection equipment (PPE), first aid kits and health benefits.

The theoretical background of this research centres on the principles of occupational safety and health (OSH), which emphasize the importance of ensuring safe and healthy working conditions for all individuals [17]. The principles emphasize the rights of workers to a safe and healthy working environment, as well as the responsibilities of employers and governments to establish OSH policies, promote prevention, ensure access to health services, and enforce compliance measures [17]. "Collection and dissemination of accurate information on hazards and hazardous materials" is recognized as a crucial element for achieving OSH [17]. This study contributes to the occupational safety and health of women in the fisheries sector by identifying the unique occupational hazards and risks they face, and by offering recommendations for collective interventions and advocacy efforts aimed at preventing and mitigating these hazards, thereby promoting the well-being of women.

## Methodology

### Study setting

**Fig 1:**
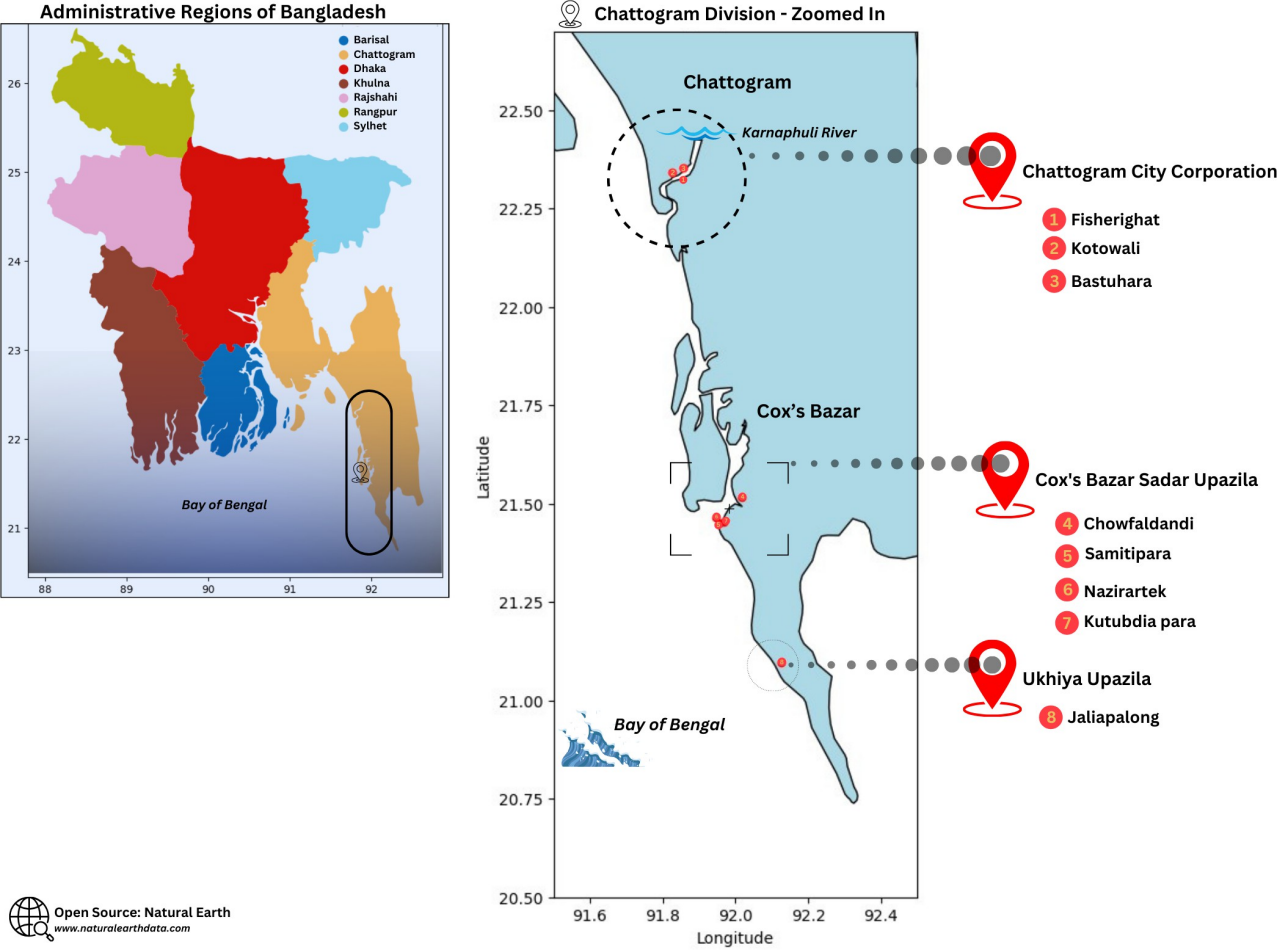
Map of the study location in Bangladesh, with a zoomed-in view of the Chattogram division. Red dots indicate the main study locations. This figure is adapted for illustrative purposes and is not identical to the original image. Map illustration adapted from Natural Earth (www.naturalearth.com).

The study was conducted among fishing communities in Cox’s Bazar and Chattogram districts in Bangladesh. **Fig 1** shows the map and detailed breakdown of the study location.

The study sites in Cox’s Bazar are located by the Bay of Bengal and inhabited by thousands of fisherfolk communities. Livelihood in these areas is mostly tied to fishing from the sea and related fishery activities [5]. Naziratek-Samitipara area, the largest dry fish processing zone of the country, accommodates hundreds of dry fish processing trades [18]. Chowfaldandi area is also inhabited by the indigenous Rakhaine community. A traditional delicacy of the indigenous people, powdered dry fish (also known as *nappi* locally), is prepared here and exported to the indigenous populations in various districts of Bangladesh [19]. Study sites in Chattogram are located by the river Karnaphulli, where fisher colonies are engaged in fishing and fish processing [15]. The fisher communities in the study sites experience poverty, characterized by underprivileged housing conditions such as fragile and overcrowded dwellings, inadequate sanitation facilities, low-income levels and limited living resources [5, 16].

The selection of study sites in Cox’s Bazar and Chattogram for our research on Bangladesh’s fisher community was driven by their significance in the country’s fisheries sector and the diverse socio-economic contexts they represent. Cox’s Bazar, with its coastal location and thriving dry fish processing trades, offered insights into traditional and commercial fishing practices, as well as the economic contributions of indigenous communities like the Rakhaine. Meanwhile, Chattogram provided a window into riverine fisheries along the Karnaphulli River, highlighting the intersection of coastal and riverine livelihoods.

### Study design

**Fig 2:**
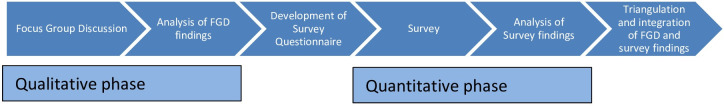
Exploratory sequential design flow of the study.

This was a cross-sectional study comprised of both qualitative and quantitative approaches aimed to explore the occupational health hazards and risks of women in the fisher communities in Chattogram and Cox’s Bazar. The information about the occupational health hazards and risks faced by women in fisher communities was gathered during the period from August 29, 2023, to October 30, 2023. The study had an exploratory sequential design (**Fig 2**), with the initial phase involving qualitative data collection and analysis preceding the subsequent quantitative phase of data collection and analysis [20]. The qualitative data collection and analysis, as described below, was done in the first place to understand the occupational setting, hazards and risks and helped in the development of the data collection tool, a structured survey questionnaire, for subsequent quantitative survey.

The qualitative study comprised five focus group discussions (FGD), two at Chattogram, two at Cox’s Bazar Sadar and one at Ukhiya Upazila. This was conducted to get an in-depth understanding of a) the socio-economic situation of the women in fisherfolk, b) potential physical safety, physical, chemical, biological and ergonomic hazards experienced by them, c) potential outcomes of the hazards to different systems of human bodies and d) existing situation of health and safety measures at workplace. A total of 48 women from fisherfolk participated in five FGDs, each participated by six to twelve individuals, as recommended by empirical studies [21–23]. All women who participated in the Focus Group Discussions (FGDs) were involved in fisheries activities as their primary occupation. Participants were purposively selected with the assistance of local volunteers. The demographic characteristics of the participants are outlined in **Table 1**.

**Table 1:** Profile of women who participated in the focus group discussions.

Age group	Type of industry		
	Dry fish processing	Raw fish processing/ marketing	Nappi/dry fish paste processing
<18 years	2	2	2
18–30 years	9	8	3
31–60 years	7	5	3
>60 years	3	3	1

A semi-structured guiding questionnaire was used for guiding the discussion, which was developed based on previous studies [7, 8, 11–13]. The questionnaire covered themes surrounding general occupational activities, physical safety hazards, ergonomics, exposure to different physical, chemical and biological hazards, workplace health safety measures and gender-specific concerns among women in the fisher communities. The guiding questionnaire is annexed as **S1 File**. The guiding questionnaire was piloted among a group of 5 fisherfolk women to ensure its consistency and applicability. The principal investigator and 5 trained research assistants facilitated the FGDs. The research assistants were rotated during the five FGDs and assigned roles such as note-taking and audio recording, local coordination, maintaining group dynamics, and observing non-verbal cues and group interactions. An interpreter fluent in the local Chittagongian language was engaged at the FGDs to eliminate the linguistic barriers. The average duration of each FGD was around 90 minutes.

The quantitative component of the study included a survey of women working in the fisheries sector using a structured questionnaire. The questionnaire form was deployed through the Kobo toolbox for data collection. The questionnaire was developed in line with the research objectives and potential occupational health hazards and illnesses explored through the qualitative study as well as the health and safety measures recommended by the Convention 188 of the International Labor Organization (ILO) [6]. The questionnaire comprised questions about a) socio-demographic profile of the participants, b) exposure to different types of physical safety, physical, chemical, biological and ergonomic hazards, as classified by Occupational Safety and Health Administration (OSHA) [24] c) potential outcomes of the occupational hazards to different systems of the human body (e.g. musculoskeletal, skin, respiratory, eye, auditory) and d) status and practice of different health and safety measures (e.g. availability and use of personal protection equipment and first aid kits, liability of farms in case of occupational injuries, provision of maternity benefits). The questionnaire was initially piloted with 10 participants, and adjustments were made as needed in the final version based on the results of the testing. Four enumerators, who were fluent in the local language, under the supervision of the research assistants were responsible for the quantitative data collection using the Kobo toolbox form. The survey questionnaire is annexed as **S2 File**.

The research assistants and enumerators were adequately trained on the research objectives and scopes, data collection instruments and procedures. The team was also oriented on obtaining informed written consent and maintaining proper privacy and confidentiality.

The study was implemented in 8 selected sites in Cox’s Bazar and Chattogram, which accommodates around 7,000 fisherfolk families. Study sites were purposively selected where diversified fishery activities are prevalent. To determine the appropriate sample size for the quantitative study, the researchers used a calculated occupational injury rate of 85% from a similar setting [16] as a reference point. Cochran’s formula was applied to calculate the sample size required for a 95% confidence level and a 5% sampling error. Initially, this calculation yielded a sample size of 191. However, the researchers decided to adjust the final sample size to 207 participants considering the potentiality of unresponsiveness. Participants were selected using simple random sampling from a local list of fisher families

### Data analysis

The qualitative data, i.e. FGD transcripts, were transcribed verbatim and manually analyzed using a thematic analysis approach [25]. This involved identifying recurring themes, patterns, and insights from the qualitative data, contributing to a richer understanding of the occupational health hazards and their impact faced by women in the fisherfolk communities. Descriptive analysis of the data collected through the structured survey questionnaire was carried out using the built-in analysis tools in Kobo Toolbox and SPSS (Statistical Package for the Social Sciences).

### Ethical consideration

All ethical guidelines have been meticulously followed throughout the research process. The Institutional Review Board (IRB) and Ethics Review Committee (ERC) of North South University in Bangladesh approved the research protocol (2023/OR-NSU/IRB/0810). All participants willingly gave written informed consent, primarily through thumbprints, and these consent documents were securely stored. The study adhered to the "no-harm" principle, ensuring no legal risks for participants. Each step was conducted following the venerable Helsinki Declaration (1964) and its 2013 revision.

## Results

### Demographic profile of the participants

The study engaged 207 women working in the fisheries sector in Cox’s Bazar and Chattogram. **Table 2** summarises the demographic profile of the participants. Nearly half of the participants (43.48%) were aged between 31 to 40 years. The majority of the participants could not sign (64.25%) and 18.36% could only sign. The ethnicity of the majority of the participants was Bengali (94.20%), and 5.80% were tribal belonging to the Rakhaine community.

**Table 2:** Socio-economic and demographic variables of the participants.

Demographic variable	Number	Percentage (N = 207)
**Age**	** **	** **
18–30 years	52	25.12%
31–40 years	90	43.48%
41–50 years	45	21.74%
51–60 years	18	8.70%
>60 years	2	0.97%
**Family type**	** **	** **
Nuclear	144	69.57%
Joint	63	30.43%
**Education**	** **	** **
Cannot sign	133	64.25%
Can only sign	38	18.36%
Upto Class 5	22	10.63%
Class 6 to Class 9	12	5.80%
SSC* Passed	2	0.97%
**Ethnicity**	** **	** **
Bengali	195	94.20%
Tribal	12	5.80%

* SSC means secondary school certificate, a nationwide examination after studying for 10 years in school.

**Fig 3:**
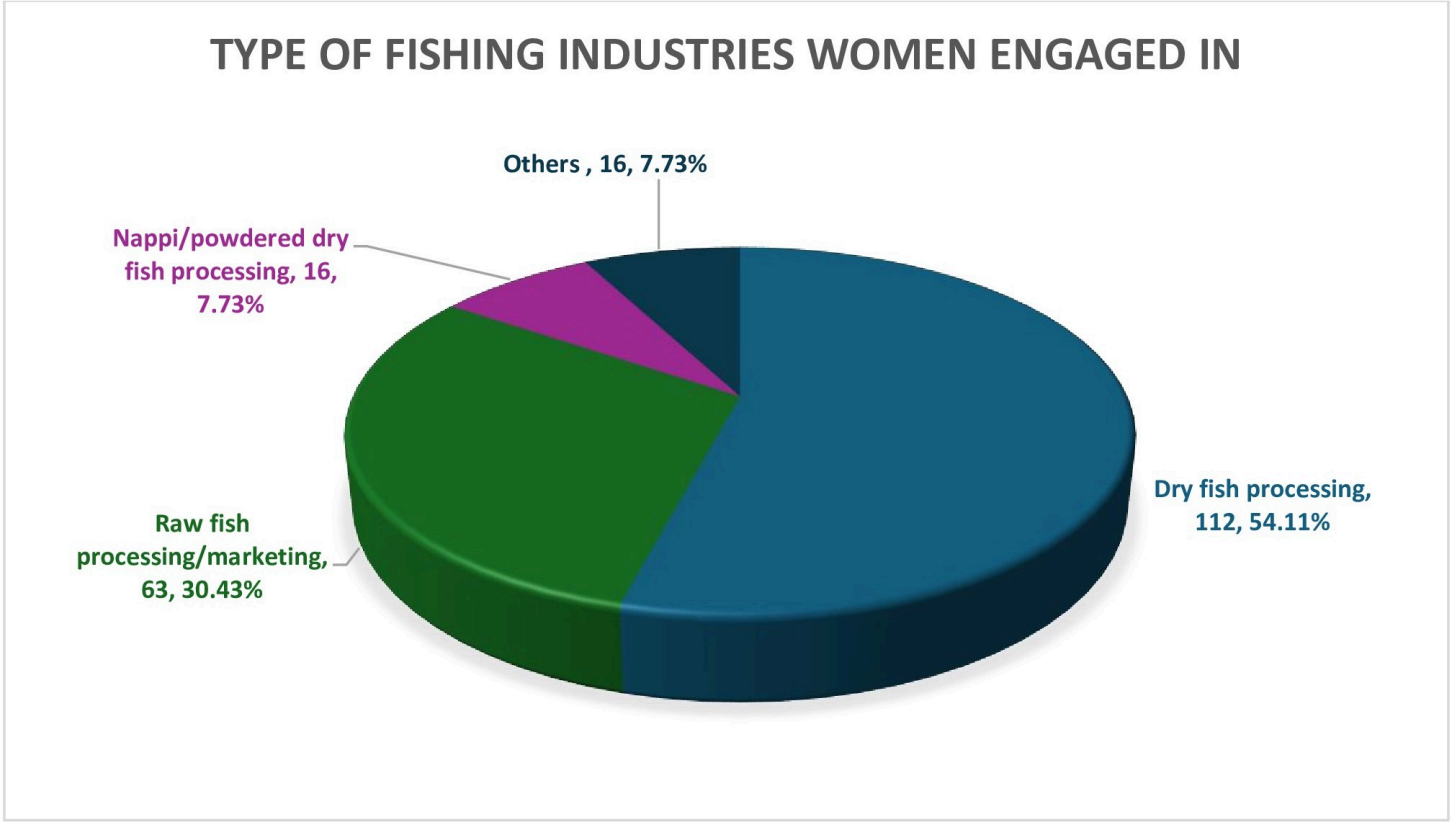
Type of fishing industries women engaged in.

More than half of the women (54.11%) were engaged in dry fish processing farms, either commercial or personal. One-third of the women (30.43%) were engaged in raw fish processing and marketing. 7.73% of the women were engaged in dry fish paste (nappi) processing and the rest were engaged in other fishery activities such as minnow or seashell collection (**Fig 3**).

**Fig 4:**
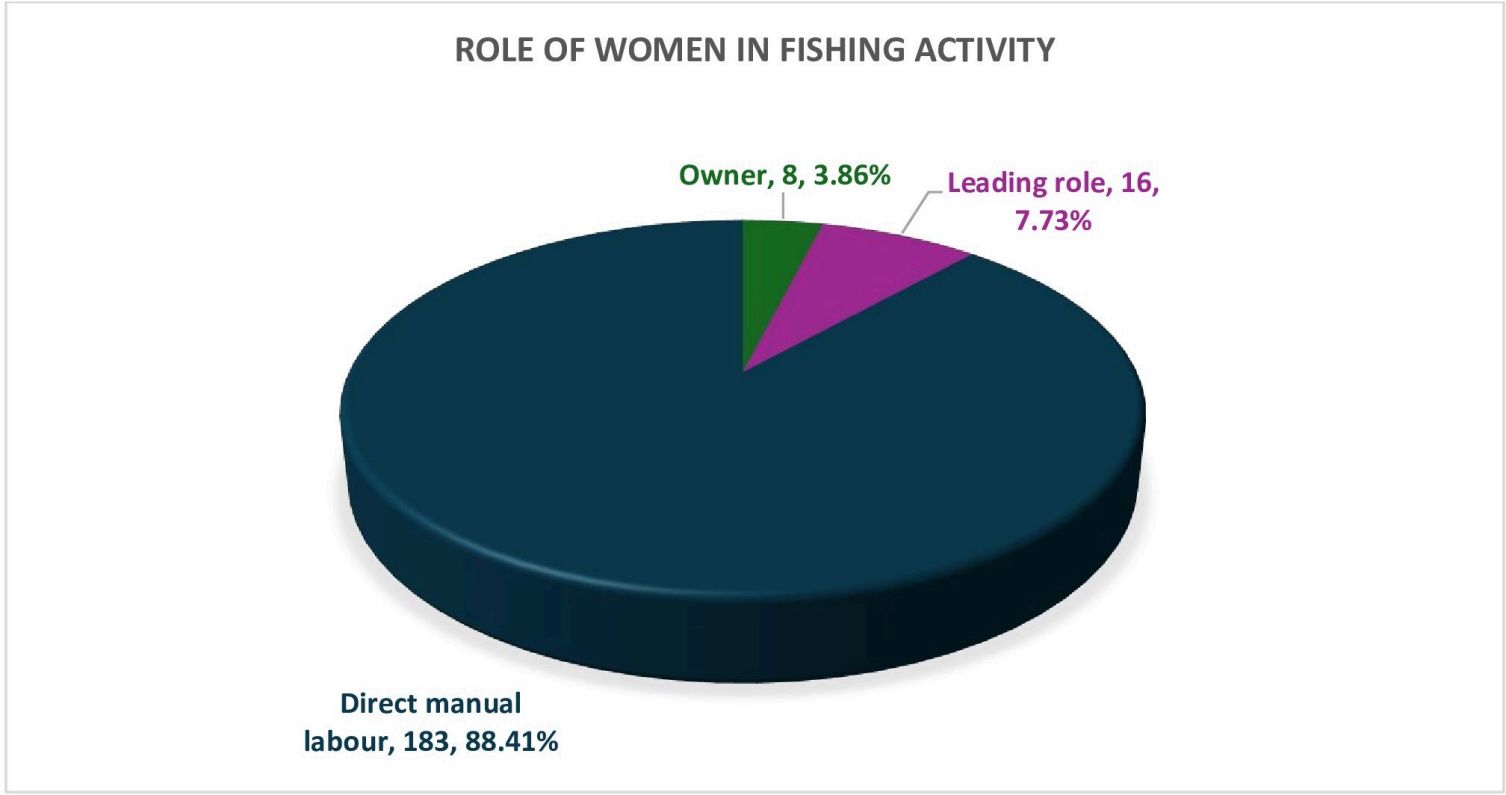
Role of women in fishing activity.

The majority of the women dedicated direct manual labour (88.41%) in their fishing industry, while only a few were owners (3.86%) or played leading roles (Majhi) (7.73%) in the fishing industry **(Fig 4)**.

When we asked about the day-to-day activities of the women in the fisher folks, we found that they were engaged in multiple fishery activities **(Table 3)**. Three-fourths of the women (73.91%) were engaged in fish sorting and grading. Around one-third of the women engage themselves in fish cutting or removing scales (34.3%), dry fish processing (32.85%) and minnow collection (32.37%) from seashores. More than a quarter of the respondents were engaged as day labourers in fishing farms (28.02%) and in seashell collection (25.12%). 17.87% of the women were found engaged in dry fish paste (*nappi*) processing. Table 3 also demonstrates that despite being engaged in three major fishing industries, women in fisher communities share overlapping day-to-day activities. Findings from our focus group discussions also revealed variations in the day-to-day activities of these women based on seasonality. For example, during off seasons when fishing is prohibited, women engage in other activities such as minnow collection and seashell collection.

**Table 3:** Daily activity chart of the participants.

Type of daily activity	Type of fishing industry				Total # of participants engaged in the activity (n = 207)	Percentage of total sample (n = 207)
	Dry fish processing (112)	Raw fish processing/marketing (63)	Nappi/powdered dry fish processing (16)	Others (16)		
Fish sorting/grading	90	59	4	0	153	73.91%
Fish cutting/removing scale	65	2	2	2	71	34.30%
Dry fish processing	64	3	1	0	68	32.85%
Minnow collection	40	13	0	14	67	32.37%
Day labourer on fishing farm	50	5	3	0	58	28.02%
Seashell collection	27	4	8	13	52	25.12%
Powdered dry fish/Nappi processing	23	0	14	0	37	17.87%
Direct fishing	13	1	1	3	18	8.70%
Selling fish/fish products	11	3	2	0	16	7.73%
Basket production	3	0	10	0	13	6.28%
Small shopkeeper	9	2	0	0	11	5.31%
Fishing net making	5	0	0	0	5	2.42%
Majhi	2	0	1	1	4	1.93%
Transporting fish/fish products	4	0	0	0	4	1.93%

### Occupational hazards

The study identified a wide range of physical safety, physical, chemical, biological and ergonomic hazards exposed by the women working in the fisheries sector. Table 4 summarises the hazards encountered.

**Table 4:** Occupational hazards for women working in the fishing industry.

Physical safety hazards		
Slippery surface	129	62%
Fishing or minnow-collecting instruments	90	44.4%
Fish cutting instruments	79	38%
Fish sting/fish bite	120	58%
Contact with fish	102	49%
Sharp stone/corals or seashells	5	2%
Basket making	2	1%
Grinding machine of Nappi	2	1%
Drifting away	2	1%
**Physical hazards**		
Sun exposure (>4 hours)	205	99%
Noise	65	31.4%
**Chemical hazards**	** **	
Chemical compounds	89	42.99%
Saltwater	191	92.27%
Salt	167	81%
**Ergonomic**	** **	
Uncomfortable body posture	188	91%
Prolonged sitting in an uncomfortable posture	174	84%
Prolonged standing	103	50%
Heavy weightlifting	108	52%
Repeated pulling/troughing or hanging	5	2%
**Biological**		
No sanitary latrine at the workplace	150	72.46%
No facility for washing hands with soap at the workplace	162	78.26%
Working long wearing wet clothes	155	74.88%

### Physical safety hazards

The participants identified a range of physical safety hazards that caused them injuries. 62% of the participants identified slippery surfaces at their workplace as a hazard which causes accidental falls and injuries. 44.4% of the participants reported fishing or minnow collecting instruments, such as nets and bamboo poles as hazards, and 38% of the participants reported fish cutting instruments, such as knives, chopper (*da*) and traditional fish knives (*boti)* as hazards causing injuries. 58% of the women suggested that they got injuries either due to fish stings or fish bites while handling the fish for sorting, grading and processing for drying. 49% reported that just contact with some species of fish might cause allergic reactions and inflammations.

FGD findings also revealed that most of the women working in the fisheries sector are to directly handle the fish for sorting, grading, cutting, descaling or drying. Those who engage in dry fish processing also need to make special knots near around gum of some fish species. Since they handle the fish without any personal protection equipment (e.g. gloves), they commonly encounter injuries from the fish spines or teeth. Often, such injury leaves with short- and long-term pores on the hands and feet of the women.

### Physical hazards

Most women in the fisheries sector worked under open sky under direct sun as 99% of the participants reported they were exposed to direct sunlight for more than 4 hours a day. 31% of the women reported that they got exposed to noise at their workplace, mostly from workplace chaos (29.5%).

### Chemical hazards

Most of the women who participated in the study reported that they had contact with salt water (90%) and salt (81%) in their day-to-day work. They encountered salt mainly during dry fish processing or dry fish paste (nappi) processing. Some women (42%) reported having contact with or being exposed to chemical compounds, especially different kinds of insecticides, during dry fish processing.

### Ergonomic hazards

The majority of the women (91%) reported that in their occupation they need to work in uncomfortable body posture. 84% of women had to sit in an uncomfortable position for a long period. FGD revealed this happens especially during collecting fish from nets, collecting minnows from the coast, sorting and grading fish, and cutting or processing fish when they have to sit on their feet without any support to the hip or back. More than half of the participating women had to lift heavy weights, especially, fish and fish products.

### Biological hazards

The study identified some biological hazards that may contribute to the transmission of infectious diseases. Nearly three-fourths of the women reported that they did not have the provision of sanitary latrines (72.46%) and handwashing with soap (78.26%) at their workplaces. Nearly the same proportion (74.88%) of the women reported that they needed to remain wearing wet clothes for a prolonged time.

Additionally, during FGDs, women from some fishing communities expressed that they did not have any specific schedule for work or rest. They need to rush whenever the boat arrives. Parallel to the economic activities, the women in the fisher communities needed to maintain household chores and take care of their children. In some communities, even during the off-fishing season, the women got engaged in non-fisheries activities, such as working as housekeepers.

### Occupational risks or health conditions

**Table 5** summarises the occupational risks in terms of illnesses and health conditions resulting from the occupational hazards.

**Table 5:** Occupational risks in terms of illnesses and health conditions.

	Number	Percentage (N = 207)
**Injury**	** **	** **
Ever encountered an injury from fishing activity	181	87.44%
Sharp Cut Injury	104	50.24%
Laceration	96	46.38%
Fracture	24	11.59%
Bruise	38	18.36%
Amputation	6	2.90%
Blunt Trauma	42	20.29%
Pores over hand and feet (Due to fish bite/sting)	63	30.43%
**Musculoskeletal disease/complaints:**		
Suffered from any kind of musculoskeletal disease/complaints	143	69.08%
Generalized body ache	95	45.89%
Back pain	109	52.66%
Neck pain	105	50.72%
Multiple joint pain	60	28.99%
Single joint pain	37	17.87%
Joint stiffness	15	7.25%
Reduced range of movement	9	4.35%
**Limb weakness**	73	35.27%
Numbness of hands or feet	36	17.39%
Fracture	10	4.83%
Deformity	1	0.48%
**Skin Problems**		
Recently suffered from any kind of skin disease or complaints?	117	56.52%
Skin allergy	87	42.03%
Skin irritation	60	28.99%
Skin redness	47	22.71%
Skin ulcers or wounds	22	10.63%
**Eye Disease/complaints**	** **	** **
Recently suffered from any type of eye complaints	110	53.14%
Eye irritation	44	21.26%
Ocular pain	48	23.19%
Red Eye	53	25.60%
Reduced vision	53	25.60%
Eye Injury	8	3.86%
**Ear disease/complaints**		
Recently suffered from any kind of ear disease or complaints	70	33.82%
Earache	10	4.83%
Itching in the ear	25	12.08%
Reduced hearing ability	27	13.04%
Vertigo	56	27.05%
**Respiratory**		
Symptoms of chronic cough (>2 weeks)	53	25.60%
Severe respiratory distress in the last 12 months	50	24.15%
Chronic respiratory discomfort or breathlessness?	29	14.01%
**Reaction/sensitivity to salt water**		
Saltwater causes physical symptoms	116	56.04%
Burning sensation in the eye	75	36.23%
Skin inflammation	64	30.92%
Itching in the ear	30	14.49%
Earache	16	7.73%
**Reaction/ sensitivity or illness due to sun exposure**		
Sun exposure caused any kind of physical symptoms	176	85.02%
Headache	146	70.53%
Skin irritation	60	28.99%
Sunburn	123	59.42%
Burning sensation in the eye	75	36.23%
**Reaction to intense foul smell**	** **	
Nausea	1	0.48%
Vomiting	34	16.43%
Vertigo	114	55.07%
Headache	101	48.79%
**History of communicable diseases in last 12 months (self-reported)**	** **	
Diarrhoea	132	63.77%
Dysentery	45	21.74%
Severe respiratory infection	11	5.31%
Flu/Viral fever	37	17.87%
Tuberculosis	8	3.86%
Scabies	108	52.17%
Tinea	106	51.21%
Helminthiasis	61	29.47%

### Injuries

87.44% of the women engaged in the fishery industry who participated in the study reported that they encountered an injury in their lifetime due to fishery activities. The injuries included sharp cut injury (50.24%), laceration (46.38%), fracture (11.59%), bruise (18.36%), amputation (2.90%) and blunt trauma injury (20.29%). Around one-third of the participants (30.43%) reported that they developed multiple pores on their hands and feet due to repeated fish bites or stings. During focus group discussions, some women also expressed that a few days after the injuries sometimes the wounds got infected.

### Musculoskeletal disease/complaints

More than two-thirds of the women (69.08%) experienced a musculoskeletal disease condition. Generalized body aches (45.89%), back pain (52.66%), neck pain (50.72%) and multiple joint pain (28.99%) were the common manifestations. Severity and chronicity of the disease varied among the participants– 24.64% reported as acute and severe, 33.82% reported acute but mild and 29.47% reported chronic and severe.

### Skin disease/symptoms

More than half of the respondents (56.5%) gave a history that they recently suffered from a skin disease or had a skin-related symptom. Skin allergy (42.03%), skin irritation (28.99%) and redness of skin (22.71%) were the common skin manifestations.

### Eye diseases/complaints

More than half of the respondents reported that they recently experienced eye-related disease symptoms or had eye diseases, such as eye irritation (21.26%), ocular pain (23.19%), red eye (25.60%) and reduced vision (25.6%).

### Ear disease/complaints

Around one-third of the responding women (33.82%) reported that they recently had an ear disease or experienced ear-related symptoms, including reduced hearing ability (13.04%) and vertigo (27.05%).

### Respiratory illnesses/symptoms

A quarter of the participants reported that they had symptoms of chronic cough for more than two weeks. 24.15% reported that they had respiratory distress in the last 12 months. 14.01% of the women complained that they had chronic respiratory discomfort or breathlessness.

### Reaction/sensitivity to salt water

56.04% of the women who participated in the study reported that exposure to saltwater caused them some kind of physical symptoms. Burning sensation of the eye (36.23%) and skin irritation (30.92%) were the common manifestations.

### Reaction/ sensitivity or illness due to sun exposure

The majority of the respondents (85.02%) informed that prolonged exposure to the sun caused some physical complaints, including headache (70.53%), skin irritation (28.99%), and eye irritation (36.23%). 59.42% of the women experienced a sunburn.

### Reaction to intense foul smell

Intense smell from fish during dry fish or dry fish paste processing, or chemical compounds causes vertigo among 55.07%, headache among 48.79% and vomiting among 16.43% of the women.

### History of communicable diseases in last 12 months

The self-reported incidence of communicable diseases was found very high among the women working in the fisheries sector. Communicable diseases experienced in the last 12 months include diarrhoea (63.77%), dysentery (21.74%), severe respiratory infection (5.31%), flu/viral fever (17.87%), tuberculosis (3.86%), scabies (52.17%), tinea (51.21%), and helminthiasis (29.47%).

### Preventive and safety measures

Overall, 78.26% of the women responded that they did not use any personal protection equipment at their workplace. **Table 6** summarises the use of different types of personal protection equipment by the women at their workplace.

**Table 6:** Use of personal protection equipment by the women working in the fishery industry (n = 207).

Use of PPE	No	Rarely	Sometimes	Often
Facemask	143	37	26	1
	(69.08%)	(17.87%)	(12.56%)	(0.48%)
Hand gloves	163	14	28	2
	(78.74%)	(6.76%)	(13.53%)	(0.97%)
Gumboot	196	9	2	0
	(94.69%)	(4.35%)	(0.97%)	(0.00%)
Raincoat/similar rain protection	124	25	34	13
	(59.90%)	(12.08%)	(16.43%)	(6.28%)
Sunglass	202	4	1	0
	(97.58%)	(1.93%)	(0.48%)	(0.00%)
Sunscreen	195	6	4	2
	(94.20%)	(2.90%)	(1.93%	(0.97%)

More than 69% of the women never used a mask in their workplace and 17.87% only used it on rare occasions. 78.74% of the women never used any hand gloves in their workplace and 6.76% used it rarely. More than 94% of the women never used gumboot. More than 94% of the women never used sunscreen or sunglasses for sun protection. 59.90% of the women never used a raincoat or similar material for rain protection, and 12.08% used it on rare occasions. As expressed by the women during FGDs, the common reasons for not using a PPE at the workplace were unavailability of PPE, discouragement by the fish farm owners, and operational inconvenience.

93.72% of the women reported that they did not have any first aid kit at their workplace which could be accessed during an injury or emergency health condition.

Participants in the focus group discussions also expressed that the fishing farms did not take any liability for treatment or care in case the workers got injured or sick due to their occupation. There were no special measures or arrangements organized at the workplace for pregnant or lactating women. Payments were made based on daily activity, there was no system in place for maternity leave or benefits.

## Discussion

This study gives in-depth insights into the occupational hazards and health risks experienced by the women actively engaged in the fisheries sector in Cox’s Bazar and Chattogram. While there are few studies available on the occupational health hazards and risks among fishermen in Bangladesh [7, 26], to the best of the authors’ knowledge, this is the first study carried out among the fisherwomen in Bangladesh revealing their occupational health hazards and risks. The study found a high occurrence of occupational hazards, health risks and disease conditions and limited availability of preventive measures among the women in the fisher communities in coastal areas of Bangladesh.

### Socio-demographic situation of the women in fisher communities

The demographic profile of our study participants reflects the distinctive characteristics of women working in the fisheries sector, highlighting alarming levels of illiteracy among fisherwomen. The literacy rate is comparatively very low among the fishermen in Bangladesh [18, 19] and due to gender-based discrimination, the rate is further low among women in the fisherfolk communities [5]. Customized awareness-raising and prevention strategies tailored to the educational status of women are imperative to address these disparities effectively. This aligns with findings from other studies in similar contexts, such as those conducted in coastal regions of India and Kenya, where educational disparities among fisherwomen were also observed [27, 28]. Additionally, a previous study conducted among fisherwomen in Cox’s Bazar and Chattogram highlighted that these women are grappling with poverty, with an annual income of only BDT 54,929, which is significantly lower than the national average [5]. Living in poverty with limited resources may exacerbate the occupational hazards faced by fisherwomen, as it can lead to a lack of access to personal protective equipment and healthcare services, and may compel them to undertake riskier tasks.

Our study revealed that women were engaged in mainly three types of fisheries industries—dry fish processing, raw fish processing and marketing and dry fish paste (*nappi*) processing, with the majority of them dedicating direct manual labour. Traditionally, women in the coastal regions of Bangladesh are not engaged in fish harvesting, which is usually seen as the occupation of men. This aligns with the social norms of the majority of the world’s small-scale fishing communities, where men are the primary producers and women get engaged in fish processing, marketing and distribution [29]. Interestingly, a significant majority of women were involved in different fishing operations at the same time, demonstrating the variety and adaptability of their positions in this sector. Common daily chores were fish sorting and grading, fish cutting or scale removal, and minnow collection illustrating the multifarious nature of their employment. Women in the fisher communities also need to get engaged in household chores and caregiving for their children [5]. Such double burden of household responsibilities and economic workload may have a significant effect on their health [30].

### Physical safety hazards and injuries

Our study identified numerous physical safety hazards leading to injuries among fisherwomen, including slippery surfaces, fishing/minnow collecting instruments, fish cutting sharp instruments, and fish stings or bites. These hazards contribute to various injuries, such as sharp-cut injuries, lacerations, fractures, and amputations. Such physical hazards and resulting injuries among fisherwomen were also evidenced in previous studies among fisherfolks of coastal states [31] and tribal communities in India [12] and Fisherfolk in Kampi Samaki, Kenya [11]. Although most of the injuries are minor and nonfatal, unable to properly treat the wound in a timely way may result in infection [32]. We found fish sting or bite is highly prevalent among the women working in fishing industries. Such injuries caused by fish species are common among men and women working in the fishery sector and reported by a large number of studies in Nigeria [8], Kenya [11] and India [12]. Additionally, we identified the risk of drifting out to sea, especially among those working near the coast, and sustaining injuries from sharp objects like stones and oysters. These findings align with prior research indicating the dangers faced by women in such environments, particularly the risk of drowning due to restrictive traditional clothing [13] and severe wounds from oyster shells [12]. Adequate safety measures, including the provision of personal protective equipment and first aid kits, are crucial to prevent physical safety hazards and mitigate the risks of injuries effectively.

### Ergonomic hazards and musculoskeletal disease conditions

The study found a high prevalence of musculoskeletal disease conditions among the women in the fishery sector including, generalized body ache, back pain, neck pain, joint pain and stiffness, reduced range of movement, muscle weakness, numbness of limbs and physical deformity. This is consistent with a study among fisherwomen in India, which found that 67% of the fisherwomen complained of musculoskeletal pain and discomfort, especially in the lower back, knee and upper back [31]. In our setting, this finding could be related to a wide range of ergonomic hazards identified by this study, such as working in uncomfortable body posture, prolonged sitting in an uncomfortable position without hip or back support and carrying heavy weight. Such uncomfortable and unsupported body posture may result in different musculoskeletal conditions, including low back pain, cervical pain and joint pain. Heavyweight carrying and walking long distances were identified as risk factors for musculoskeletal disease conditions among fisherwomen in previous studies in Kenya [11] and India [12]. Given the high prevalence of musculoskeletal disease conditions among women in the fisheries sector, interventions targeting ergonomic improvements are imperative to safeguard their health and well-being.

### Chemical hazards and potential risk to diseases/health conditions

Although the use of pesticides in the dry fish industry is strictly prohibited by the law in Bangladesh, some women informed that they get exposed to pesticides in their dry fish farms. Occupational exposure to pesticides can be associated with respiratory health problems, including asthma, chronic obstructive pulmonary disease (COPD) and lung cancer [33]. While exploring the statistical association between pesticide exposure and clinical conditions was not within the scope of this research, the study did find a high incidence of self-reported respiratory complaints among fisherwomen, indicating a need for further investigation into the association between chemical exposure and respiratory diseases. Our study highlights that a majority of women in fisher communities have frequent contact with salt water and salt in their daily activities and alarmingly, more than half of the women reported experiencing physical symptoms, such as eye irritation, skin irritation, ear itching, and earache, in reaction to the salt water. Moreover, we observed a high incidence of skin diseases among women in fisher communities, including skin allergy, irritation, ulceration, and wounds. Exposure to salt or salt water could be a potential factor for such conditions, as supported by recent research indicating that sodium chloride could contribute to the progression of skin allergies [34]. Furthermore, exposure to certain fish species may exacerbate skin irritation and allergic reactions [35]. A research among fisherwomen in India also argued that brackish water, jellyfish, and algal bloom may also contribute to skin irritation, rashes, and psoriasis among fisherwomen [12]. Therefore, there is a crucial need to mitigate the exposure to and impact of salt and salt water among the women in the fisher communities. Further research is warranted to elucidate the underlying mechanisms and establish statistical associations between saltwater exposure and skin-related clinical conditions among fisherwomen.

### Physical hazards and skin and eye diseases

The study found that nearly all (99%) of the women got exposed to direct sunlight for more than 4 hours a day and the majority of them reported some kind of reaction or sensitivity to sun exposure, which included headache, skin irritation, sunburn and eye irritation. Ultraviolet radiation of sun rays may result in acute sunburn and chronic skin changes, including skin cancer [36]. Sunburn among fisherwomen was also reported as a health concern in Kenya [11]. Kyei et al identified seawater and sunrays as ocular hazards causing ocular irritation, tearing, red eyes and blurred vision [37]. We also found a high incidence of eye-related diseases or symptoms among the women in fisherfolk, which include eye irritation, ocular pain, red eye and reduced vision. Further research is warranted to investigate whether there is an association between prolonged exposure to the sun to these conditions. To address the significant health risks associated with prolonged sun exposure among women in the fisher community, implementing measures such as providing protective eyewear and promoting sun-safe practices is crucial for safeguarding their ocular and overall health.

### Biological hazards and communicable diseases

Fisher folks in Chattogram and Cox’s Bazar live in overcrowded settings with fragile shelter and compromised water, sanitation and hygienic practices. Our study found that more than three-fourths of the women in the fisherfolk did not have the provision of sanitary latrines and handwashing with soap at their workplaces. All these factors can contribute to a high burden of communicable diseases among the fisherfolk community. This could be evidenced by the fact, as found in our study, of a very high incidence of self-reported communicable diseases, especially, diarrhoea, dysentery, flue/viral fever, respiratory infection, tuberculosis, skin infections and helminthiasis among the women in fisher communities. Our study revealed that a significant proportion of women were to wear wet clothes for extended periods due to their occupational activities, such as fishing and fish processing. This practice, coupled with the hot and humid climate of Bangladesh, particularly during the summer, creates an environment conducive to the development of tinea [38]. This explains the potential reason for the high prevalence of self-reported cases of tinea among the participants over the past 12 months. Although, the reported incidents of communicable diseases were not clinically verified, intensified health promotion and risk communication efforts are recommended both at the community level and at the workplace to improve hygienic behaviour and practices.

### Health safety measures

Many of the hazards could be mitigated by the use of personal protective equipment, such as gloves, masks, sunglasses, sunscreen and gumboots [11, 16]. Protective gloves prevent injuries from physical hazards like knives and fish spines, as well as chemical hazards such as salt, while eye goggles shield the eyes from sunlight. However, we have found majority of the women in the fisher communities did not use any personal protection equipment at their workplace. While the use of gloves could prevent many of the injuries associated with fish bites or sting and sharp instruments, commonly available heavy-duty gloves may not be feasible for fisherwomen since these are not suitable to use for fine work carried out by the women during fish processing. Therefore, personal protection equipment should be innovated or customized considering the local needs, context and requirements. For example, PPE designed for fisherwomen should be waterproof to withstand frequent water exposure, lightweight and breathable for comfort in hot and humid conditions and customized to allow flexible handling of fish. Moreover, the fisherwomen should be trained and mobilized to use personal protection equipment for their health and safety. Finally, we have found that the first aid kits were grossly unavailable at the workplace, with which injuries could be managed early and risk of wound infection could be prevented. Strong advocacy is needed to influence the fish industry players to make personal protection equipment and first aid kits available at the workplace.

### Limitations of the study

The study reported the hazards and health conditions based on the experiences shared by the participants. The conditions were not clinically validated. However, the author tried to mitigate the response bias by triangulation of qualitative and quantitative data by integration and comparison and appropriate training of the enumerators on case definitions of clinical conditions. The study was also only descriptive and did not have the component of analyzing the clinical correlation. Another limitation of the study was it did not include the mental health and psychosocial aspects of the participants.

## Conclusion and recommendations

Women in the fisher communities in Bangladesh are engaged in a wide range of activities in the fisheries sector. Our study found a high occurrence of occupational hazards experienced by the women, including physical safety, physical, chemical, ergonomic and biological hazards. Due to the unavailability of adequate preventive and safety measures, the hazards are resulting in a wide range of health risks and disease conditions among the women in the fisher communities, including injuries, musculoskeletal disorders, skin diseases, eye diseases, respiratory diseases and communicable diseases. However, their workplaces are pervasively missing with the availability of personal protection equipment, first aid kits and health and safety benefits. Notwithstanding the adversities, the women continue to work hard in the fishing industry, even though their efforts are merely acknowledged in society. The research highlights the need for further investigation into the underlying mechanisms and statistical associations between certain hazards and risks, such as exposure to chemicals and respiratory diseases, prolonged sun exposure and eye-related diseases, and factors contributing to infectious diseases among women in fisher communities. Utilizing established policy directives, notably the National Occupational Safety and Health (OSH) policy, we emphasize the coordinated implementation of the following recommendations. This collaborative effort involving government bodies, development partners, fisheries sector stakeholders, and community representatives will significantly enhance the health and well-being of women in fisher communities.

1. Training and Awareness: Develop comprehensive training programs tailored to the needs of fisherwomen, focusing on hazard identification, risk assessment, and safety protocols. Collaboration is needed among relevant government departments, local government institutions, NGOs, and community leaders to organize regular training and awareness sessions and disseminate promotional materials.
2. Innovative Procedures: Collaborate with fisheries sector experts to identify and implement innovative procedures for the safe handling of fish and associated instruments. This should involve developing guidelines and protocols tailored to the unique challenges faced by women in the industry.
3. Ergonomic Solutions: Invest in engineering and ergonomic innovations to address ergonomic hazards, such as proper sitting arrangements and ergonomic tools for heavy lifting. This involves engaging with fisheries sector leaders and experts to identify and implement practical solutions.
4. Access to Essential Resources: Ensure the availability of essential resources such as first aid kits and personal protective equipment (PPE) at all workplaces. A focal person should be assigned to each fishery community to oversee inventory management and training on proper use.
5. Advocacy: Facilitate advocacy targeting fish industry owners to improve workplace safety, hygiene, and ergonomic conditions, restrict chemical hazards, and ensure health checkups and maternal benefits for women in the fisheries sector.

## Supporting information

S1 Dataset Dataset of the study is attached as a reference. (XLSX)

S1 File Guidance questionnaire for focus group discussion. (PDF)

S2 File Questionnaire for the survey of the women. (PDF)
