# Supplementary material for: Occupational hazards and risks among the women in fisher communities in Cox’s Bazar and Chattogram, Bangladesh
Source: PLoS One. 2024 Jul 19;19(7):e0297400. doi: 10.1371/journal.pone.0297400 (PMC11259271; doi:10.1371/journal.pone.0297400)
Supplement: S1 File — (PDF) [file pone.0297400.s002.pdf]

Occupational hazards and risks among the women in fisher communities in Cox's Bazar and Chattogram

Qualitative Questionnaire

Introduction:
Fishery is one of the oldest and most prevalent occupations in Cox's bazar and Chattogram coastal communities. Fishery engages a wide range of occupational activities, including production, harvesting, processing, handling, storage, and transportation. Traditionally, men and women have different roles, while men are mostly engaged in harvesting, women are mostly engaged in fish processing. Therefore, women may experience distinctive hazards and risks in their occupation, which is vital to reveal to ensure a safe work environment for them. Moreover, it is also essential to understand the measures in place to prevent occupational hazards and risks, such as personal safety training and personal protection equipment, for proper planning of preventive strategies.

Consent Note: As part of this survey, with your consent we will be asking you some questions about your profession. The interview will take approximately 90 to 120 minutes. All information provided during the interview will remain confidential, and you are free to choose whether to answer the questions. Feel free to ask any questions about the interview before agreeing to participate. There are no risks associated with this interview, and you can stop the interview at any time. Your name will not be included in the report. The information you provide will be valuable for the health of individuals involved in fishing. Thank you. With your consent, we would like to begin this interview based on the information provided.

Demographic Information:

1. Age:
2. Occupation:
3. Location:
4. Educational Background:

1. General occupation:

a. What is your typical occupation? What activities you do at your occupation? What is your working hour?

b. What other fishing activities you perform? What do you do during the off season?

2. Physical and safety hazards:

a. Tell us something about your workplace. What are the workflows and processes? What equipment and materials are there? Is there any equipment or material that can cause harm to your health?

b. What kind of physical hazards you face at your workplace that may acutely threated your life? [Hints: injury from heavy, dangerous, and unguarded equipment]

c. Have you or your relative/colleagues experienced any injuries. What are the common causes of injuries at your workplace? [Hints: reflect on instruments, gears, fish handling, fish bite, type of fish etc.]

d. How often you expose to sun? Do you or your relative/colleague face any skin irritability or other skin conditions due to sun exposure? Have you heard anyone developed skin cancers?

e. Do you often suffer from any eye problem in relation to your occupation? What could be the possible causes? Do you get allergy or eye sensitivity to salt water, sun or any other factors?

f. If the injury is related to fish bite or fish handling, can you mention the species of the fish/es and how the injury happened?

g. Do you suffer from any skin problem in relation to your occupation? What could be the possible causes? Do you get allergy or skin sensitivity to salt water or fish?

3. **Ergonomics:**
   a. Do you/your co-workers have experienced any musculoskeletal problem? What are those problems? Is there any occupational factor/s that you think contributing to this disorder? [hints: falls on slippery wet surface, lifting heavy weight]

4. **Chemical:**
   a. Are there any chemical compounds that are used at your workplace? Does any of this cause any health-related problem to you?

   b. If you are engaged in drying fish or fish processing, what are chemical hazards you are being exposed to? How do you handled that? What harm it may cause you?

   c. Do you suffer from any skin problem in relation to your occupation? What could be the possible causes? Do you get allergy or skin sensitivity to salt water or fish?

   d. Is there any way you expose to vapor or smoke? Do you face any discomfort or health problem due to it?

   e. What measures are taken to prevent these chemical hazards?

5. **Biological:**
   a. What are the fishes or insects that you may get expose at your workplace? What kind of health problem it may cause? Explain us the sufferings.

   b. Do you have sanitary latrine? How do you practice your personal hygiene? How often your or your colleagues suffer from diarrhea or other water borne diseases (e.g. dysentery, typhoid).

   c. What common infectious diseases you catch at your workplace? How are these related to your workplace?

   d. What are the common skin diseases you/your colleague's encounter? Give us some ideas on infectious skin conditions but also other skin ulcers (e.g. foot, hands). Do you think is there any reason behind this?

   e. Do you/your colleagues face any respiratory diseases due to the type of your occupation?

   f. What measures are taken to prevent these biological hazards?

6. **Workplace health safety:**
   a. What is the minimum age of work for your occupation?

   b. How do you arrange your food and drinking water?

   c. What safety and social protection measures available for the maternal and reproductive health? Is there any provision for maternity leave? Is there any convenient system in place for pregnant and lactating mothers? Is there breast-feeding corner available at the workplace?

   d. Did you/your co-workers use any personal protective equipment to protect you from the hazards?

e. Do you have or bring first aid kit at your workplace? What are the medicines and supplies you have in this kit? Is anyone in your team are trained on using the first aid it?

f. Did you get any kind of formal guidance or training from experts on safe handling of fishing gears or maintaining health safety at your workplace?

g. Is there any system in place for periodic medical checkup arranged by the employer? Did you require any medical clearance certificate for working in this job?

h. Is there any mechanism in place for reporting or investigation of accidents for prevention of future incidents?

i. How do you seek medical care in case you became severely sick or get a serious injury?

7. Gender:

a) Is there disproportionately or differently girls and women impacted by different occupational hazards? How and why?

b) What are the occupational hazards girls and women exclusively face due to their gender perspectives?

8. Fatality at workplace:

a) How prevalent is the fatality among the fisher women caused by their occupational factors? What are the common reasons and/or factors?
