# Supplementary material for: Occupational hazards and risks among the women in fisher communities in Cox’s Bazar and Chattogram, Bangladesh
Source: PLoS One. 2024 Jul 19;19(7):e0297400. doi: 10.1371/journal.pone.0297400 (PMC11259271; doi:10.1371/journal.pone.0297400)
Supplement: S2 File — (PDF) [file pone.0297400.s003.pdf]

Occupational hazards and risks among the women in fisher communities in Cox’s Bazar and Chattogram

Quantitative Questionnaire

Introduction:

Fishery is one of the oldest and most prevalent occupations in Cox's bazar and Chattogram coastal communities. Fishery engages a wide range of occupational activities, including production, harvesting, processing, handling, storage, and transportation. Traditionally, men and women have different roles, while men are mostly engaged in harvesting, women are mostly engaged in fish processing. Therefore, women may experience distinctive hazards and risks in their occupation, which is vital to reveal to ensure a safe work environment for them. Moreover, it is also essential to understand the measures in place to prevent occupational hazards and risks, such as personal safety training and personal protection equipment, for proper planning of preventive strategies.

Consent Note: As part of this survey, with your consent we will be asking you some questions about your profession. The interview will take approximately 90 to 120 minutes. All information provided during the interview will remain confidential, and you are free to choose whether to answer the questions. Feel free to ask any questions about the interview before agreeing to participate. There are no risks associated with this interview, and you can stop the interview at any time. Your name will not be included in the report. The information you provide will be valuable for the health of individuals involved in fishing. Thank you. With your consent, we would like to begin this interview based on the information provided.

Demographic Information:

1.1 ID (A/C/S/T, 001):

1.2 Age (years):

1.3 District:
- Cox's Bazar
- Chattogram

1.4 Upazila:
- Chittagong City Corporation
- Cox's Bazar Sadar
- Ukhiya
- Other (specify)

1.5 Union:
- Fisherighat
- Kotowali
- Bastuhara
- Chowfaldandi
- Samiti para
- Naziratek
- Kutubdia para
- Jaliapalong
- Other (specify)

1.6 Type of family:
- Nuclear
- Joint

1.7 Education:
- Cannot sign
- Can sign only
- Up to Class V
- Class VI-IX
- SSC Pass
- HSC Pass
- Bachelor or above

1.8 Caste
- Bengali
- Tribal

1.9 What is your occupation?
- Dry fish processing
- Raw fish processing/marketing
- Nappi/ dry fish paste processing
- Others (Specify)

1.10 Which work you mainly involved in?
- Fishing
- Dry fish farm work
- Fish grading
- Fish cutting
- Daily labour in fishery
- Net making
- Powder dry fish (Nappi) processing
- Selling fish/dry fish
- Transporting fish/dry fish
- Boat owner
- Marketing
- Fish wholesale activity (aarot)
- Ice processing
- Basket making
- Small shop
- Other fishery activity (Specify)

1.11 What is your type of work ?
- Personal
- Commercial

1.12 What is your main role in your occupation?
- Owner
- Leading (e.g. Majhi)
- Direct manual labor

2.1 Physical hazards:

2.1.1 How often do you get exposed to the sun?
a) No exposure or < 1 hour
b) 1 – 3 hours a day
c) 4 – 6 hours a day
d) > 6 hours a day

2.1.2 Do you get exposed to salt water?
a) No
b) Rarely
c) Sometimes
d) Often

2.1.3 Does your occupation engage you in lifting heavy weights?
e) No
f) Rarely
g) Sometimes
h) Often

2.1.4 Does your occupation engage you in uncomfortable posture (e.g. sitting, standing, pulling etc.)?
i) No
j) Rarely
k) Sometimes
l) Often

2.1.5 Do you get exposed to any chemicals at your work?
a) No
b) Rarely
c) Sometimes
d) Often

2.1.6 If yes, what kind of chemical?

a) Chemical compounds
b) Salt water
c) Salt
d) Other: Specify

2.1.7 Do you get exposure to noise?
a) Yes
b) No

2.1.8 If yes, what produces the noise?
a) Engine room
b) Generator of workplace
c) Crowding of the place
d) Other:

2.1.9 Did you experience any of the following hazards/factors?
a) Fall on a slippery wet surface
b) Getting caught in winches
c) Lifting heavy weight
d) Prolonged sitting in an uncomfortable posture
e) Prolonged standing
f) Repeated pulling, throwing or hanging
g) Other relevant hazards: __________________________

2.2 Chemical/Biological/Ergonomic hazards

2.2.1 Do you have an arrangement of sanitary latrines at your workplace/vessel?
a) Yes
b) No

2.2.2 Do you use soap and water to wash your hands after passing stool at your workplace/vessel?
a) Yes
b) No

2.2.3 Do you have to work in damp clothing for a long period of time?
a) Yes
b) No

2.2.4 Do you use any personal protective equipment at your workplace?
a) Yes
b) No

2.2.5 Do you use face mask at your work?
a) No
b) Rarely
c) Sometimes
d) Often

2.2.6 Do you use gloves at your work?
e) No
f) Rarely
g) Sometimes
h) Often

2.2.7 Do you use gumboots at your work?
i) No
j) Rarely
k) Sometimes
l) Often

3. Potential outcomes to the different human system

3.1 Did you recently suffer from any eye illness?

a) Yes
b) No

3.2 If yes, what kind of eye illness have you developed? [multiple answers possible]
a) Eye irritation (including discomfort, itchiness or dryness)
b) Eye pain
c) Red eye
d) Reduced vision
e) Cataract/cloudy lens
f) Eye injury
g) Others: please specify

3.3 Did you recently suffer from any skin illness?
a) Yes
b) No

3.4 If yes, what kind of skin illness you developed? [multiple answer possible]
a) Allergic reaction
b) Skin irritation
c) Skin rash
d) Ulcer
e) Skin cancer
f) Other: specify

3.5 Do you have sensitivity to salt water?
a) Yes
b) No

3.6 If yes, what sensitivity do you experience?
a) Eye irritation
b) Skin irritation
c) Ear itching

d) Earache
e) Other (please mention):

3.7 Do you develop any sensitivity or illness due to sun exposure?
a) Yes
b) No

3.8 If yes, which type of sensitivity or illness? (multiple selection)
a) Headache
b) Skin irritation
c) Sunburn
d) Eye irritation
e) Other: Please specify

3.9 Do you have any ear problem?
a) Yes
b) No

3.10 If yes, what type of ear problem do/did you experience?
a) Earache
b) Ear itching
c) Reduced hearing
d) Vertigo
e) Other: please specify

3.11 Do you have any musculoskeletal disease/illness?
a) Yes
b) No

3.12 If yes, what type of musculoskeletal symptoms you experience?
a) General body ache
b) Low back pain
c) Cervical pain
d) Multiple joint pain
e) Single joint pain
f) Joint stiffness
g) Reduced range of motion
h) Weakness of limbs
i) Numbness
j) Fracture
k) Deformity
l) Other:

3.13 Does this symptom/s acute or chronic in the course?
a) Acute
b) Chronic

3.14 What communicable diseases have you had in the last 12 months?
a) Diarrhoea
b) Dysentry
c) Acute respiratory infection
d) Tuberculosis (if diagnosed)
e) Scabies
f) Ring worm
g) Worm infestation

3.15 Do you have the symptom of chronic cough (>2 weeks)?
a) Yes
b) No

3.16 Did you ever have severe respiratory distress in last 12 months?
a) Yes
b) No

3.17 Do you have any of the symptoms of chronic respiratory discomfort or breathlessness?
a) Yes
b) No

3.18 Have any of your family member/relative/neighbor died due to any of the hazards associated with their fishery activity?

3.19 If yes, what was the reason:
____
____.

4. Health and safety measures

4.1 Have you ever had injury due to the fishing activity?
  a) Yes
  b) No

4.2 What type of injury did you have?
  a) Sharp cut
  b) Laceration
  c) Fracture
  d) Bruise
  e) Amputation
  f) Blunt trauma
  g) Other, specify

4.3 How did you had the injury?
  a) From fishing gears
  b) From fish stings
  c) From fish cutting devices

4.4 What symptoms did you have while you suffered from the injury?
  a) Pain
  b) Swelling
  c) Bleeding
  d) Ulcer
  e) Abscess/pus formation
  f) Deformity
  g) Chronic immobility/disability

4.5 What measure you have taken to treat the condition?
  a) Self-treatment
  b) Local pharmacy
  c) Traditional treatment
  d) Formal doctor/hospital

4.6 Do you use raincoat/rain protective gear at your work?
  a. No
  b. Rarely
  c. Sometimes
  d. Often

4.7 Do you use sunglasses/eye goggles at your workplace?
  a. No
  b. Rarely
  c. Sometimes
  d. Often

4.8 Do you use sunscreen creams at your workplace?
  a. No
  b. Rarely
  c. Sometimes
  d. Often
